# Micropeptide MIAC inhibits the tumor progression by interacting with AQP2 and inhibiting EREG/EGFR signaling in renal cell carcinoma

**DOI:** 10.1186/s12943-022-01654-1

**Published:** 2022-09-19

**Authors:** Mengwei Li, Guangxiang Liu, Xinrong Jin, Hongqian Guo, Sarra Setrerrahmane, Xindi Xu, Tiantian Li, Yunfei Lin, Hanmei Xu

**Affiliations:** 1 The Engineering Research Center of Synthetic Peptide Drug Discovery and Evaluation of Jiangsu Province, China Pharmaceutical University, Nanjing, 210009 China; 2 State Key Laboratory of Natural Medicines, Ministry of Education, China Pharmaceutical University, Nanjing, 210009 China; 3 Department of Urology, Drum Tower Hospital, Medical School of Nanjing University, Institute of Urology, Nanjing University, Nanjing, 210008 Jiangsu China; 4 NANJING ANJI BIOTECHNOLOGY CO. LTD, Nanjing, 210033 China

**Keywords:** Non-coding RNA, Micropeptides, Renal cell carcinoma, MIAC, Aquaporin 2, EREG/EGFR pathway

## Abstract

**Background:**

Although, micropeptides encoded by non-coding RNA have been shown to have an important role in a variety of tumors processes, there have been no reports on micropeptide in renal cell carcinoma (RCC). Based on the micropeptide MIAC (micropeptide inhibiting actin cytoskeleton) discovered and named in the previous work, this study screened its tumor spectrum, and explored its mechanism of action and potential diagnosis and treatment value in the occurrence and development of renal carcinoma.

**Methods:**

The clinical significance of MIAC in RCC was explored by bioinformatics analysis through high-throughput RNA-seq data from 530 patients with kidney renal clear cell carcinoma (KIRC) in the TCGA database, and the detection of clinical samples of 70 cases of kidney cancer. In vitro and in vivo experiments to determine the role of MIAC in renal carcinoma cell growth and metastasis; High-throughput transcriptomics, western blotting, immunoprecipitation, molecular docking, affinity experiments, and Streptavidin pulldown experiments identify MIAC direct binding protein and key regulatory pathways.

**Results:**

The analysis of 600 renal carcinoma samples from different sources revealed that the expression level of MIAC is significantly decreased, and corelated with the prognosis and clinical stage of tumors in patients with renal carcinoma. Overexpression of MIAC in renal carcinoma cells can significantly inhibit the proliferation and migration ability, promote apoptosis of renal carcinoma cells, and affect the distribution of cells at various stages. After knocking down MIAC, the trend is reversed. In vivo experiments have found that MIAC overexpression inhibit the growth and metastasis of RCC, while the synthetized MIAC peptides can significantly inhibit the occurrence and development of RCC in vitro and in vivo. Further mechanistic studies have demonstrated that MIAC directly bind to AQP2 protein, inhibit EREG/EGFR expression and activate downstream pathways PI3K/AKT and MAPK to achieve anti-tumor effects.

**Conclusions:**

This study revealed for the first time the tumor suppressor potential of the lncRNA-encoded micropeptide MIAC in RCC, which inhibits the activation of the EREG/EGFR signaling pathway by direct binding to AQP2 protein, thereby inhibiting renal carcinoma progression and metastasis. This result emphasizes that the micropeptide MIAC can provide a new strategy for the diagnosis and treatment of RCC.

**Supplementary Information:**

The online version contains supplementary material available at 10.1186/s12943-022-01654-1.

## Introduction

With the advancement of multi-omics next-generation sequencing (such as ribosomal mapping and protein/peptide omics) and computational biology, continuous evidences [1–3] revealed the existence of small open reading frame (sORF) within non-coding RNAs (such as lncRNA, circRNA, pri-miRNA), 5'UTR, 3'UTR, antisense transcripts, intergeneric non-coding regions, etc. These sORFs can produce functional micropeptides participating in the regulation of biological processes such as muscle development [4], energy metabolism [5], immune regulation [6], and tumor development [7, 8]. Huang et al. [9] found that the micropeptide HOXB-AS3 encoded by lncRNA HOXB-AS3 inhibits the growth of colon cancer (CRC), and Polycarpou-Schwarz et al. [10] discovered the micropeptide CASIMO1 (cancer-associated small integral membrane open reading frame 1), which has an oncogenic effect. Its high expression promotes the proliferation of breast cancer cells. In addition, tumor-associated micropeptides have also been found in liver cancer, rectal cancer, glioma, acute myeloid leukemia (AML) and other cancers, such as SMIM30 [11], MPM (micropeptide in mitochondria) [12], ASAP (ATP synthase-associated peptide) [13], APPLE (a peptide located in ER) [14].

In our previous work, high-throughput RNA-seq sequencing data analysis, CRISPR/cas9 gene editing, proteomics technics were applied to prove that lncRNA AC025154.2 encode a new endogenous micropeptide in head and neck squamous cell carcinoma (HNSCC) [15], named MIAC (Micropeptide Inhibiting Actin Cytoskeleton). In vitro functional experiments and mechanism studies have proved that the interaction of MIAC and AQP2 proteins inhibits the expression of Spet2 and ITGB4, and then regulates the expression of actin cytoskeleton, thereby inhibiting the occurrence and development of HNSCC. Further, TCGA database to screen the tumor spectrum of MIAC differential expression, it was found that MIAC was also differently expressed in five other tumors (including kidney cancer, thyroid cancer, prostate cancer, lung adenocarcinoma, and colon adenocarcinoma), but the specific manifestations were different. Among them, MIAC expression level in RCC tissues is significantly lower than that of normal adjacent tissues. This differential expression is significantly related to the prognosis of patients. Functionally, the effect of chemically synthetized MIAC peptides in inhibiting the proliferation and migration of RCC cells was found to be very significant, suggesting that MIAC may have important diagnostic and therapeutic value in RCC, but the specific mechanism of action still not clear.

In this paper we combined clinical sample analysis, in vitro and in vivo functional experiments, high-throughput transcriptomics, western blotting, immunoprecipitation, molecular docking, affinity assessment to elucidate the specific functions and molecular mechanisms of MIAC in RCC, and explore the potential value of MIAC in the clinical diagnosis, prognosis and treatment of RCC, so as to provide an important reference for the discovery of new strategies for kidney cancer diagnosis and treatment.

## Materials and methods

### TCGA database analysis

The RNA seq data and clinical pathologic information (Table S1) of 530 KIRC tumors and 72 normal renal tissues adjacent to tumors was obtained through the TCGA data portal (https://portal.gdc.cancer.gov/). The obtained reads (counts) were normalized to their library sizes and transcript length (RPKM normalization). To compare the percentage survival between different MIAC and AQP2 expression groups, Kaplan–Meier survival curves were generated.

### Clinical specimens

All the clinical tumor specimens were obtained from Nanjing Drum Tower Hospital, the Affiliated Hospital of Nanjing University Medical School. The formalin-fixed and paraffin-embedded (FFPE) specimens from 7 patients and the frozen tumor tissues from 63 patients who underwent surgery. Tissues were obtained after patients’ written consent under a protocol approved by the institution’s Institutional Review Board.

### Cell lines and cell culture

Human renal clear cell carcinoma 786-O and A498 cell lines, embryonic kidney (HEK) 293T cell line were obtained from the American Type Culture Collection. 786-O cells were cultured in RPMI-1640 medium (Life Technologies, Carlsbad, CA, USA) supplemented with 10% FBS (Bioind, USA). A498 cells were cultured in MEM (Life Technologies, Carlsbad, CA, USA) supplemented with 10% FBS. HEK-293T cells were cultured in DMEM (Life Technologies, Carlsbad, CA, USA) supplemented with 10% FBS.

### Cell transfection and vector construction

Lentiviral vectors (pLenti-CMV-GFP-Puro, addgene) harbouring the cDNA sequence were co-transfected with psPAX2 (Addgene) and PMD2.G (Addgene) into HEK-293T cells. Cell transfections with plasmids or siRNAs were performed using Lipofectamine 3000 (Invitrogen) or GP-transfect-mate (GenePharma, Shanghai, China), respectively, following the manufacturer’s instructions. The stable cell lines were established by infecting cells with lentiviruses that expressed the target gene, followed by selection with puromycin. All the siRNAs were obtained from GenePharma and the sequences are listed in Table S2.

### Quantitative real-time PCR (qRT-PCR)

Total RNA was isolated using TRIZOL reagent (Invitrogen) according to the manufacturer’s instructions. And cDNA was synthesized using High-Capacity cDNA Reverse Transcription Kit (G592, abm, Canada) following the manufacturer’s protocol. The mRNA levels were detected by SYBR Green real-time PCR Master Mix (G891, abm, Canada) and performed on ABI QuantStudio 3 Real-Time PCR System (Applied Biosystems). The mRNA expression was normalized by the expression of GAPDH and relative expression levels were calculated using the 2^-ΔΔCT method in cell and tissue lysates. Primers are shown in Supplementary Table S3.

### Protein extracting and Western blotting analysis

Total protein was extracted from cell samples using RIPA lysis buffer (Beyotime Biotechnology) supplemented with Thermo Scientific Halt Protease Inhibitor Cocktail (Thermo Fisher Scientific). Protein extracts were resolved by SDS-PAGE and then electrophoretically transferred onto a PVDF membrane (EMD Millipore). The membrane was incubated for 2 h in blocking buffer (1 × TBST containing 5% non-fat milk), and then was incubated at 4℃ overnight with following primary antibodies: MIAC (abmart, 1:1000), AQP2 (abcam, ab199975), EREG (Cell Signaling, 12,048), EGFR (Proteintech, 18,986–1-AP), Phospho-EGFR (Abmart, T55232), mTOR(Abmart, T55306), p-mTOR (Abmart, T56571), Akt (Abmart, T55561), Phospho-Akt (Abmart, T40067), ERK1/2 (Abmart, T40071), Phospho-ERK1/2 (Abmart, TP56192), GAPDH (Proteintech, 60,004–1-Ig), HA (Abmart, M20003S). The immunocomplexes were subsequently incubated with the HRP-conjugated secondary antibodies, and detected with the Western Blot Detection kit (Beyotime Biotechnology) and TANON-5200 system (Tanon, Shanghai, P.R. China). The densitometric ratio of protein bands was calculated by ImageJ program.

### Co-Immunoprecipitation (Co-IP)

Cell extraction was performed using IP Lysis Buffer (Beyotime Biotechnology) supplemented with Thermo Scientific Halt Protease Inhibitor Cocktail, and then incubated with primary antibody overnight at 4℃. Next day, the antibody-bound protein of interest in lysis buffer was incubated with 40 μl of Protein A Agarose Beads (9863, Cell Signaling Technology). After three washes with Wash Buffer (0.5 M Tris–HCl pH 7.4, 1.5 M NaCl), protein-bound beads were mixed with 5 × loading buffer (Yeasen, Shanghai, P.R. China) and boiled for 10 min at 95℃. The samples were then stored at -20℃ or ready for western blotting analysis.

### Cell proliferation assay

Cell proliferation assays were performed using Cell Counting Kit-8 (Hanbio). Briefly, cells were seeded in 96 well plate with 100 μl of complete medium per well. CCK-8 reagent was added into each well at 4 h prior to measurement. Absorbance at 450 nm was measured by a Multiskan Plate Reader (Thermo Fisher Scientific) at indicated time points.

CellTrace Violet Cell Proliferation kit used for in vitro labeling of different cells to trace multiple generations using dye dilution by Flow cytometry (BECKMAN COULTER, CytoFlex). Specifically, the indicated cells were suspended at a density of 1 × 10^6^ cells mL^−1^ in PBS/0.1% bovine serum albumin (BSA) in preparation for cell labeling. CellTrace Violet (Molecular Probes, Thermo Fisher Scientific, C34557) was prepared at twice the final concentration in PBS/0.1% BSA, and a volume equivalent to each cell suspension was added to each tube immediately prior to incubation in a 37℃ water bath for 20 min. The staining was stopped with completed medium, washed twice for 5 min, and cells were detected by FACS analysis and placed into culture.

### Transwell migration assays

The RCC cells were harvested and seeded with serum-free medium into the upper chambers at 4 × 10^4^ cells/well, and the bottom chambers containing culture media and 10% FBS as a chemoattractant, and then incubated for 48 h at 37℃, successfully translocated cells were fixed by ice-cold methanol and stained with 2% crystal violet, and imaged using an inverted microscope (Olympus, IX53P1F).

### Cell apoptosis and cell cycle detection

Apoptosis assay in cells was performed using an Annexin V-PE/7-AAD Apoptosis Assay Kit (KeyGEN BioTECH) according to manufacturer’s instructions. Briefly, A498 cells were harvested and incubated with Annexin V-PE and 7-AAD for 15 min. Apoptotic cells populations were analyzed by FACS system. The cell cycle phase distribution were determined by propidium iodide (KeyGEN BioTECH) staining. Briefly, A498 cells were harvested and and fixed with 70% ice-cold ethanol for at least 2 h. After centrifugation at 1000 rpm for 5 min, the cells were washed and resuspended with 0.5 mL PI/RNase buffer for 30 min in the dark. Subsequently, the cell cycle distribution was tested by FACS system.

### In vivo animal models

Male BALB/c-nude mice (6–8 weeks old) were maintained under pathogen-free conditions in the Laboratory Animal Center of China Pharmaceutical University and subsequent procedures were performed in accordance with the institutional ethical guidelines for animal experiments. Briefly, to detect the effect of MIAC on the tumourigenicity of RCC cells, 5 × 10^6^ A498 cells stably with MIAC overexpression or mock cells were subcutaneously injected into the mice, respectively. The length and width of the mice tumors were measured every three days and the tumor volume was calculated using the following formula 0.5 × length × width^2^.

In the in vivo metastasis model, 3 × 10^6^ A498 cells stably with MIAC overexpression or mock cells were injected into the tail vein of mice, tumor progression was monitored twice a week through IVIS Spectrum living imaging system (Perkin Elmer) for four weeks. At the end point of the experiment, mice were humanely euthanized.

For efficacy of chemosynthetic MIAC peptide studies, 5 × 10^6^ A498 cells were subcutaneously injected into the mice according to the above protocol. When the volume of xenograft reached 100 mm^3^, mice were randomly assigned into seven groups and intravenous (IV) injections with different dosage of MIAC peptides (5 mg/kg, 10 mg/kg, 15 mg/kg) or saline every day for four weeks, and intragastric injections with sunitinib (Selleck, 20 mg/kg, 40 mg/kg) or Axitinib (Selleck, 30 mg/kg) every day for four weeks. The length and width of the mice tumors were measured every three days and the tumor volume was calculated using the above formula.

### Molecular Docking

The 3D structures of the peptide MIAC was generated in PEP-FOLD3 (https://bioserv.rpbs.univ-paris-diderot.fr/services/PEP-FOLD3/). PEP-FOLD is a de novo approach aimed at predicting peptide structures from amino acid sequences [16]. This method, based on structural alphabet SA letters to describe the conformations of four consecutive residues, couples the predicted series of SA letters to a greedy algorithm and a coarse-grained force field. The 3D structure of the protein AQP2 were downloaded from RCSB Protein Data Bank (PDB ID: 4OJ2). Protein–protein docking in HDOCK [17] was used to predict the binding affinity with MIAC and AQP2. ClusPro [18] is a web-server that based on a hybrid algorithm of template-based modeling and ab initio free docking. The docked structures and interface residues were analyzed using MOE 2019.1.

### Streptavidin–biotin pull-down assay

HEK293T cells transfected with plasmids including recombinant HA-AQP2 and different AQP2 mutation for 72 h, cells were lysed in ice-cold lysis buffer as the above protein extracting protocol. The soluble fractions from cell lysates were incubated with synthesized biotin-conjugated peptide MIAC (GL Biochem, Shanghai) overnight at 4℃. Then, Streptavidin beads were added the mix and rotated for 2 h at 4℃, followed by washing with PBST buffer three times. The binding components were eluted by boiling with 1 × Loading Buffer and were analysed by western blot for HA.

### Microscale thermophoresis (MST) assay

MST assay was performed to detect binding interactions of peptide MIAC and AQP2 protein as previously described [19]. Chemosynthetic peptide MIAC (NANJING ANJI BIOTECHNOLOGY CO. LTD) was labeled by CY5 fluorescence (Xi’an ruixi Biological Technology Co., Ltd) via the sulfhydryl group on the cysteine. HEK293 cells were used to express the membrane protein Flag-Mcherry-AQP2. With the existence of the TEV cleavage site, the Flag-Mcherry tag was excised by a TEV protease. The obtained AQP2 protein was identified by liquid phase and mass spectrometry. The labeled MIAC with AQP2 protein were incubated 5 min at room temperature, and reaction mixtures were enclosed in premium-coated glass capillaries and loaded into the instrument (Monolith NT.115, NanoTemper, Germany). Kd values were determined using the NanoTemper MO. Affinity Analysis tool as described.

### SPR (Surface Plasmon Resonance) assay

A BIAcore T200 instrument (GE Healthcare) was used to detect binding interactions using a direct binding assay format [20]. Prior to activation, the research grade CM5 chip surface was preconditioned using 100 μL injections of HBS-EP buffer at a flow rate of 30 μL/min. AQP2 (10 μg/mL) was immobilized on the sensor surface using standard amine coupling with 10 mM NaAc, pH 4.0 buffer. Peptide were injected over the active protein and reference surface with at least 30 s association and dissociation times. Surface regeneration was achieved using dissociation for a time period allowing the response to return to baseline. Control injections of different concentration of MIAC peptide to allow monitoring of the functionality of the protein surface. SPR equilibrium binding data, consisting of Req values from 5 points concentration series, were analyzed by fitting a simple 1:1 binding to yield Rmax and Kd values using Biacore T200 Evaluation Software.

### Statistical analysis

Experimental data were statistically analyzed using Prism 9.0 software program (GraphPad Software Inc., San Diego, CA, USA). The statistical signifcance of diferences was evaluated by two-tailed Student’s t test or two-way ANOVA, derived from triplicate samples of at least three independent experiments. For Kaplan–Meier analysis the log rank (Mantel-Cox) test was performed. *P < 0.05 was considered statistically signifcant.

## Results

### MIAC is down-expressed in renal cancer tissue and is associated with overall survival and tumor stage.

**Fig. 1:**
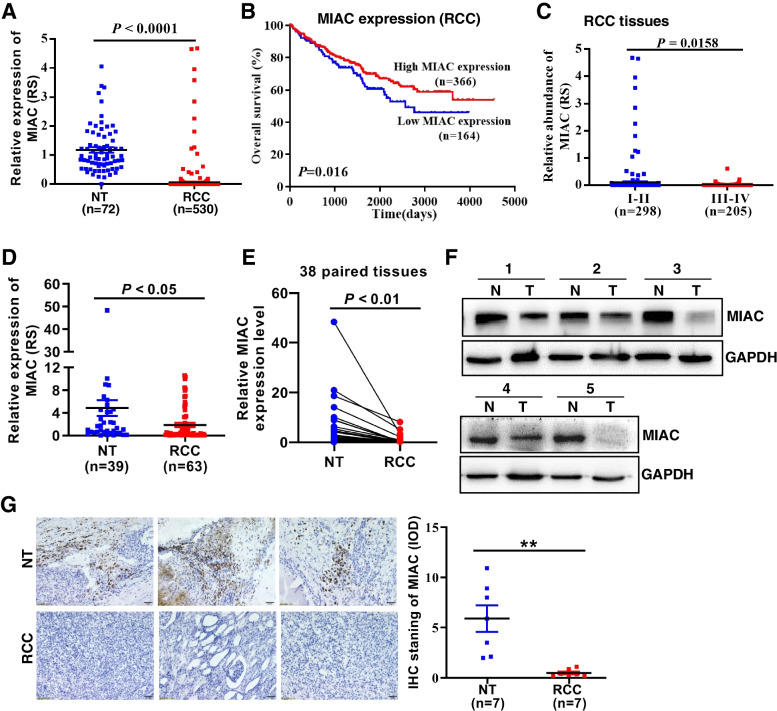
MIAC is downregulated in RCC tissue. **A** Relative expression of MIAC in KIRC samples from the TCGA database. **B** Kaplan–Meier analysis of the correlation between MIAC levels and overall survival in RCC samples. **C** Relative expression of MIAC in early and late stage of KIRC samples. **D, E** The relative expression levels of MIAC in RCC tissues and the adjacent noncancerous tissues were determined by RT-qPCR. **F, G** The protein expression of MIAC in RCC samples by Western blot and IHC assay. *P < 0.05, **P < 0.01, ***P < 0.001

The tumor spectrum with differential MIAC expression was screened through the TCGA database, and it was found that the expression of MIAC in renal clear cell carcinoma was significantly lower than that in adjacent tissues (Fig. 1A); Kaplan–Meier survival analysis showed that the overall survival rate of KIRC patients with high MIAC expression was significantly higher in patients with low MIAC expression (Fig. 1B); the correlation between MIAC expression and TNM tumor stage of patients was analyzed, and it was found that the expression of MIAC in patients with early stage I-II renal cancer was significantly higher than that in patients with advanced stage III-IV (Fig. 1C), indicating that the expression of MIAC is related to the malignancy and survival of renal cancer. At the same time, 63 clinical samples from patients with renal cancer and 39 normal tissues adjacent to the tumor were collected. The results of qPCR, WB and immunohistochemistry showed that MIAC was significantly lower expressed in renal cancer tissues compared with normal tissues (Fig. 1D-G), suggesting that micropeptide MIAC has potential clinical diagnostic and prognostic value in renal carcinoma.

### Micropeptide MIAC can inhibit the occurrence and development of renal cancer in vitro and in vivo.

**Fig. 2:**
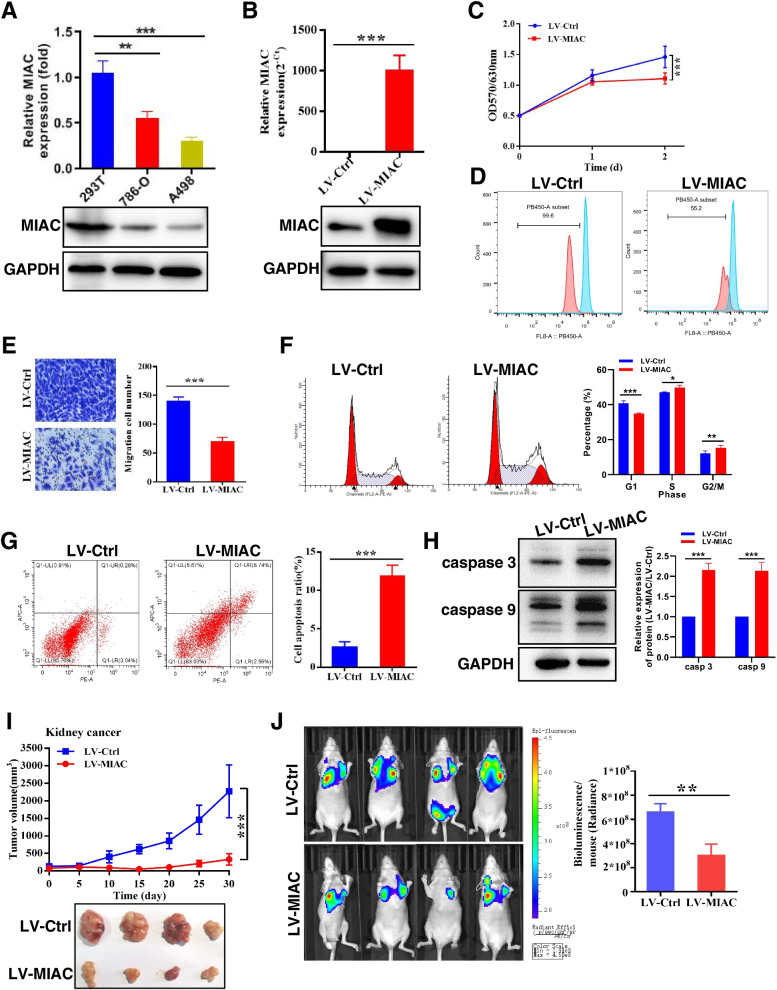
MIAC expression inhibits the growth and metastasis of RCC in vitro and in vivo. **A** The relative expression of MIAC in normal kidney cell and RCC cell lines. **B** Verification of overexpressed MIAC in A498 cells. **C, D** MIAC inhibits RCC cell proliferation by CCK8 and cell Trace CFSE assays. **E** MIAC inhibits RCC cell migration. **F** MIAC promotes RCC cell cycle arrest. **G, H** MIAC promotes RCC cell apoptosis. **I** Tumor volume in the mouse xenografts injected with A498 cells after the stable overexpression of MIAC (*n* = 4). **J** The lung metastasis of the tumor is detected by intravital imaging (*n* = 4). *P < 0.05, **P < 0.01, ***P < 0.001

Based on the clinical significance of MIAC in RCC, we explored its specific role in renal carcinoma through in vitro and in vivo functional experiments. First, a lentiviral vector was used to construct a stably transfected RCC cell line A498 overexpressing MIAC (Fig. 2A, B). CCK8 and CellTrace™ CFSE cell proliferation experiments found that overexpression of MIAC could significantly inhibit cell proliferation (Fig. 2C, D). Transwell cell migration assay found that overexpression of MIAC could significantly inhibit the migration ability of cells (Fig. 2E). Flow cytometry was analyzed that overexpression of MIAC caused cell cycle arrest in S and G2 phase (Fig. 2F). AV/PI staining and WB experiments showed that endogenous overexpression of MIAC promoted cell apoptosis (Fig. 2G, H). Furthermore, knockdown of MIAC (Fig. S1A, B) significantly promotes RCC cell proliferation (Fig. S1C) and migration (Fig. S1D), promotes RCC cell cycle arrest in G1 phase (Fig. S1E) and inhibits cell apoptosis (Fig. S1F). We further explored the effect of MIAC on tumor growth and metastasis of renal cancer cells in vivo. The results of subcutaneous xenograft experiments in nude mice found that overexpression of MIAC could significantly inhibit the tumor growth of renal cancer cells (Fig. 2I). The distal tail vein metastasis experiment showed that MIAC could significantly inhibit the in vivo lung metastasis of renal cancer cells (Fig. 2J). The above data together confirmed the tumor suppressive effect of MIAC in RCC.

### MIAC directly binds to AQP2 protein

**Fig. 3:**
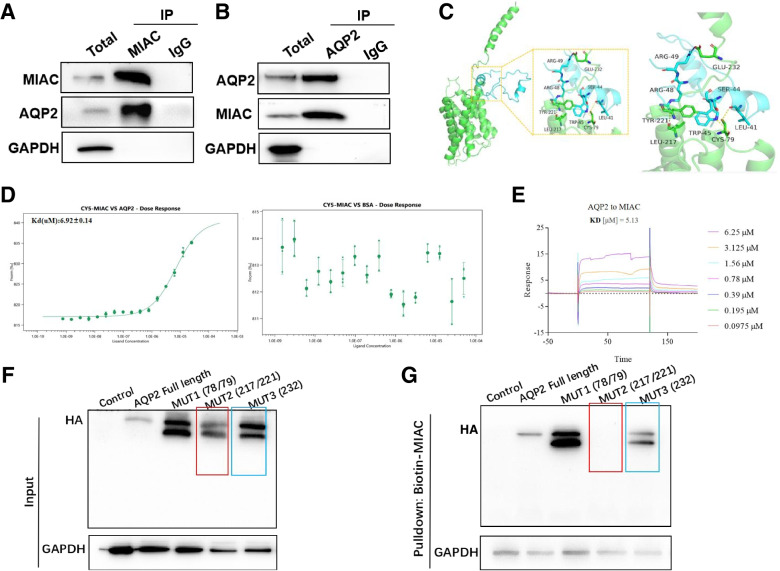
MIAC directly binds to AQP2. Immunoprecipitation (IP) and IB with anti-MIAC (**A**) or anti-AQP2 (**B**). **C** Molecular docking analysis of the MIAC and AQP2. MST (**D**) and SPR (**E**) analysis of the direct intearction between MIAC and AQP2. **F, G** Biotin-MIAC pulldown with HA-tagged AQP2 or site-directed mutants based on the molecular docking analysis in 293 T

According to previous studies, we demonstrated by co-immunoprecipitation and yeast two-hybrid experiments that the micropeptide MIAC can directly bind to AQP2 protein, regulate the actin cytoskeleton and inhibit the growth and metastasis of head and neck squamous cell carcinoma cells [15]. In this study, we wanted to explore whether the combined effect of these two molecules also exists in renal cancer. The co-immunoprecipitation experiments of renal cancer cells demonstrated that MIAC and AQP2 proteins bound to each other (Fig. 3A, B). 

 We used molecular docking to explore the direct binding of MIAC and AQP2, and the results showed that the binding sites of micropeptide MIAC mainly included L41, S44, W45, R48, R49 amino acid residues, and the binding sites of AQP2 protein included G78, C79, L217, Y221, E232 amino acid residues, via salt bridges, hydrogen bonds, and hydrophobic interactions (Fig. 3C, Table S4). Further, microscale thermophoresis (MST) and Surface plasmon resonance (SPR) binding affinity experiments were carried out using chemically synthesized MIAC polypeptide and eukaryotic expression and purified AQP2 protein, respectively. The MST results showed that the KD(M) value of direct binding between the two was 6.92E-06 (Fig. 3D), SPR results showed that the KD(M) value of the direct binding between the two was 5.13E-06 (Fig. 3E), which both confirmed that the micropeptide MIAC had a strong binding affinity to the AQP2 protein.

In order to further verify the accuracy of molecular docking and explore the key amino acid sites that MIAC binds to AQP2 protein, we performed alanine substitutions on the five amino acid sites (G78A, C79A, L217A, Y221A, E232A) that might be bound to AQP2 protein to construct recombinant plasmids (Fig. S2A), and transfected different recombinant plasmids into 293 T cells (Fig. S2B), the cell lysate was co-incubated with MIAC-biotin peptide (Fig. S2C, D) for streptavidin pulldown experiment. The results showed that Y221, L217, and E232 amino acid residues on AQP2 protein were important sites for binding to MIAC (Fig. 3F, G).

### AQP2 protein is lowly expressed in renal cancer tissue and can inhibit the proliferation and migration of renal cancer cells

**Fig. 4:**
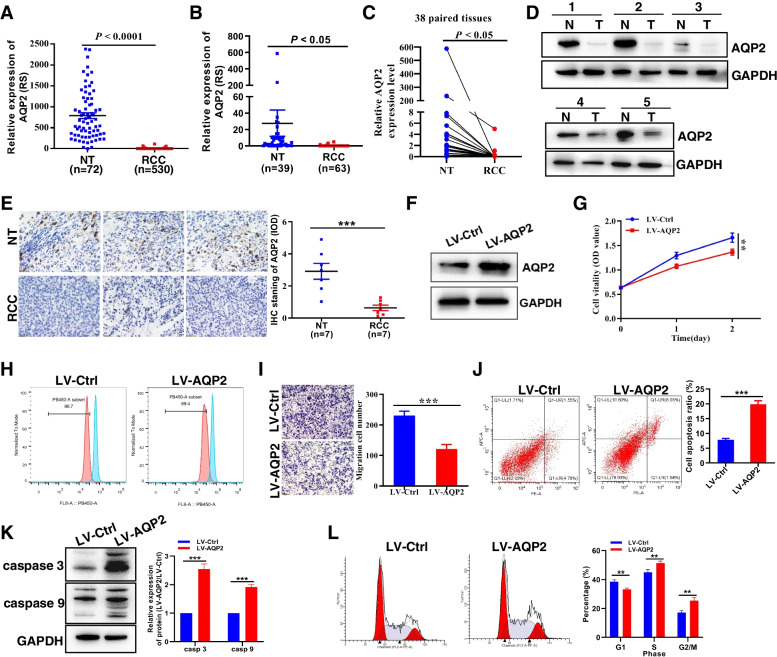
AQP2 is downregulated in RCC tissue and inbihits RCC cell proliferation and migration. **A** Relative expression of AQP2 in RCC samples from the TCGA database. **B, C** The relative expression levels of AQP2 in RCC tissues and the adjacent noncancerous tissues were determined by RT-qPCR. **D, E** The protein expression of AQP2 in RCC samples by Western blot and IHC assay. **F** Verification of overexpressed AQP2 in A498 cells. AQP2 inhibits RCC cell growth (**G, H**) and migration (**I**). AQP2 promotes RCC cell apoptosis (**J, K**) and cell cycle arrest (**L**). *P < 0.05, **P < 0.01, ***P < 0.001

As one of the members of the aquaporin family, AQP2 is an antidiuretic hormone-sensitive aquaporin [21], which is mainly expressed in kidney tissue. The initial study was involved in congenital nephrogenic diabetes insipidus, diabetes or heart disease [22]. Recent studies used public database analysis have found that AQP2 protein is related to the diagnosis and prognosis of breast cancer [23], ovarian cancer [24], glioblastoma [25], and renal cancer [26]. And our previous work found that AQP2 is highly expressed in HNSCC patients and can promote the HNSCC cell proliferation and migration [15], but the specific role in renal cancer is unclear. Combined with TCGA database analysis (Fig. 4A) and clinical renal cancer sample detection (Fig. 4B-E), it was confirmed that AQP2 protein was lowly expressed in renal cancer tissue, but the AQP2 expression is not associated with tumor stage (Fig. S3A) and overall survival (Fig. S3B). We further explored the specific role of AQP2 in renal cancer through functional experiments. CCK8 and CellTrace™ CFSE cell proliferation assays found that overexpression of AQP2 (Fig. 4F) could significantly inhibit cell proliferation (Fig. 4G, H), Transwell cell migration assay found that overexpression of AQP2 could significantly inhibit cell migration (Fig. 4I), AV/PI staining and WB experiments showed that endogenous overexpression of AQP2 promoted apoptosis (Fig. 4J, K), and flow cytometry analysis of overexpression of AQP2 affected cell cycle arrest in S and G2 phase (Fig. 4L). Conversely, knockdown of AQP2 (Fig. S3C, D) significantly increased RCC cell proliferation (Fig. S3E) and migration (Fig. S3F), promotes RCC cell cycle arrest in G1 phase (Fig. S3G). The above data prove that AQP2 is a new protein involved in regulating the occurrence and development of renal cancer.

### MIAC inhibits EREG/EGFR expression and downstream signaling pathway activation

**Fig. 5:**
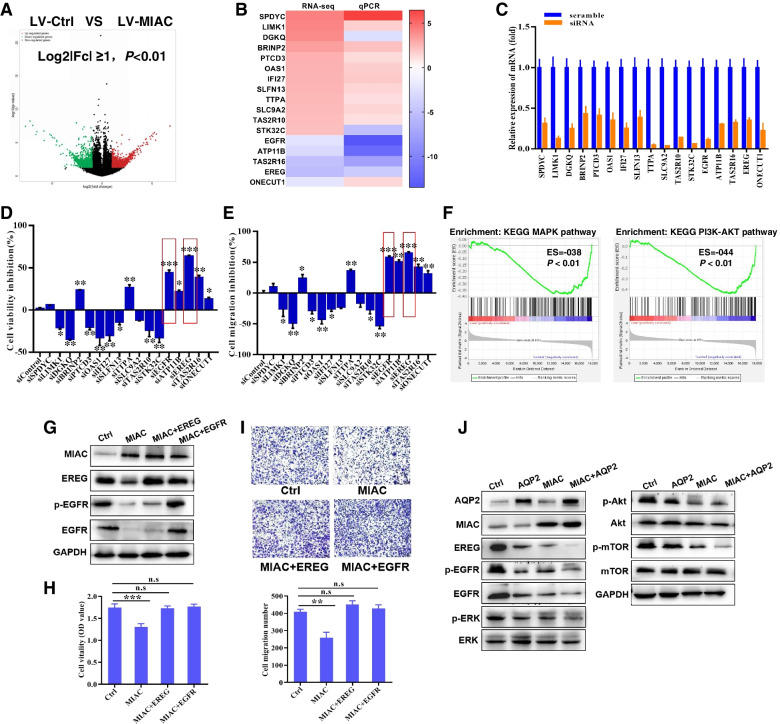
MIAC inhibits the EREG/EGFR signalling. **A** Volcano map drawn based on all differentially expressed genes after overexpressing MIAC cells. **B** qPCR verification of top down-regulated or up-regulated genes after overexpression of MIAC. **C** qRT-PCR to verify the interference efficiency of siRNA targeting candidate genes. The inhibition rate of cell proliferation (**D**) or migration (**E**) after different siRNA interference. **F** The GESA enrichment of RNA-seq analysis of MIAC overexpression. **G** Overexpression of MIAC downregulated EREG/EGFR expression. MIAC abrogates the effect of EREG or EGFR on RCC cell proliferation (**H**) and migration (**I**). MIAC interacts AQP2 and inhibits the EREG/EGFR signalling (**J**). *P < 0.05, **P < 0.01, ***P < 0.001

In order to elucidate the specific molecular mechanism by which micropeptide MIAC inhibits the occurrence and development of renal cancer, we performed high-throughput transcriptome sequencing of MIAC-overexpressing renal cancer cells and control cells, and analyzed all genes in the sequencing data with confidence intervals and differential expression folds. Screening was performed (Log2|Fc|≥ 1, *P* < 0.05), and 1768 differentially expressed genes were obtained. Compared with the control group, 847 genes were significantly up-regulated and 921 genes were significantly down-regulated in the MIAC overexpression group (Fig. 5A). To further explore the key genes regulated by MIAC, 17 genes with Log2|Fc|≥ 2 were subsequently verified and functionally screened. These included 5 genes down-regulated after MIAC overexpression (EGFR, ATP11B, EREG, TAS2R16, ONECUT1), 12 up-regulated genes after MIAC overexpression (SPDYC, LIMK1, DGKQ, BRINP2, PTCD3, OAS1, IFI27, SLFN13, TTPA, SLC9A2, TAS2R1, STK32C), the results verified by qRT-PCR were basically consistent with the sequencing results (Fig. 5B). After knocking down these genes by RNAi interference technology (Fig. 5C), combined with CCK8 cell proliferation assay and transwell cell migration assay screening (Fig. 5D, E), it was found that knocked down the epidermal growth factor receptor (EGFR) and its ligand epiregulin (EREG) have the most significant inhibitory effect on the function of renal cancer cells. At the same time, the specific signaling pathways involved in differentially expressed genes were analyzed through the GESA analysis, and it was found that the classic downstream PI3K/AKT pathways and and MAPK pathways of EREG/EGFR were significantly enriched after MIAC overexpression (Fig. 5F), so the regulation of MIAC on EREG/EGFR expression and downstream signaling pathways was further studied in the future.

WB experiments found that overexpression of MIAC significantly down-regulated the protein expressions of EGFR and EREG, while overexpression of EGFR or EREG could counteract the inhibitory effects of MIAC on EREG, EGFR expression and phosphorylated EGFR levels (Fig. 5G). Cell proliferation and cell migration experiments both demonstrated that EREG and EGFR were able to alleviate the inhibitory effect of MIAC on RCC cell function (Fig. 5H, I). Simultaneous overexpression of AQP2 protein and MIAC micropeptide can significantly down-regulate the expression of EREG and EGFR, and inhibiting the activation of downstream signaling pathways PI3K/AKT and RAS/ERK (Fig. 5J), but there is no regulatory relationship between AQP2 and MIAC. This indicates that MIAC directly binds to AQP2, and finally plays a role in inhibiting the occurrence and development of renal cancer by inhibiting the expression of EREG and EGFR and the activation of downstream signaling pathways.

### Chemically synthesized MIAC polypeptide can inhibit renal cancer in vitro and in vivo

**Fig. 6:**
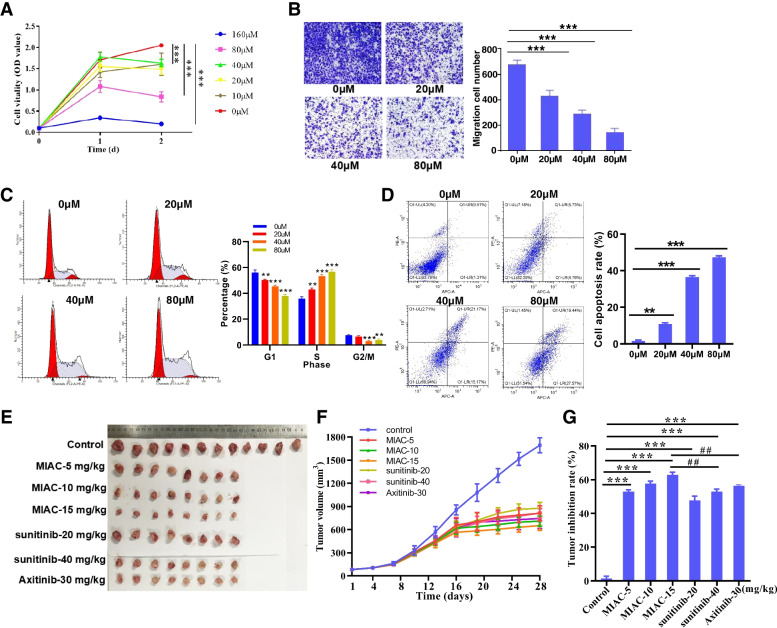
The therapeutic effects of chemosynthetic MIAC peptide on RCC in vitro and in vivo. The inhibitory activity of chemosynthetic MIAC peptide on RCC cell proliferation (**A**) and cell migration (**B**). Flow cytometry indicated that the MIAC inhibits cell cycle progression of RCC cells (**C**). Flow cytometry assays showed that the MIAC accelerated the apoptosis of RCC cells (**D**). **E** Gross image of subcutaneous tumors with different dosage of MIAC and positive drug (sunitinib/Axitinib). **F** Tumor growth curve of differnt groups. **G** Analysis of tumor inhibition rate of MIAC exogenous peptides and positive drugs after administration. *P < 0.05, **P < 0.01, ***P < 0.001

Polypeptide drugs have become one of the important directions of new drug research and development in the world because of their simple spatial structure, low immunogenicity, low toxicity and side effects, and high product purity [27]. In view of the significant antitumor effect and small molecular weight of endogenous MIAC micropeptide, this study attempted to investigate whether it has antitumor activity in renal cancer by chemical synthesis of MIAC peptide. First, the purity and molecular weight of MIAC polypeptides were determined by high performance liquid chromatography (HPLC) and mass spectrometry (LC–MS) to meet the requirements of subsequent functions (Fig. S4A, B). In vitro functional experiments showed that MIAC polypeptide dose-dependently inhibited the proliferation (Fig. 6A) and migration (Fig. 6B) of renal cancer cells, promoted S phase and G2 phase arrest (Fig. 6C) and cell apoptosis (Fig. 6D). The in vivo administration experiment of the subcutaneous transplanted tumor model showed that MIAC polypeptide can significantly inhibit the growth of renal cancer cells in vivo, and the tumor inhibitory effect at the dose of 15 mg/kg is better than that of the positive drugs sunitinib and axitinib (Fig. 6E-G). The above data indicate that MIAC polypeptide has potential as a therapeutic drug for renal cancer.

## Discussion

Renal Cell Carcinoma (RCC) is a malignant tumor originating from renal parenchymal urinary tubule epithelium [28, 29], accounting for 2%-3% of adult malignant tumors, and the most common is clear-cell RCC (ccRCC). According to GLOBOCAN statistics, there is 431,000 new cases of renal cancer and approximately 179,000 associated deaths globally every year, making renal cancer one of the most common fatal tumors in the world [30]. Most cases of renal cancer are asymptomatic at the early stage, and most of them are localized diseases, with a 5–10 years survival rate of > 90%. However, high-risk renal cancer is known to have blood-borne metastasis, and patients undergoing clinical treatment for metastatic RCC are prone to tolerance and resistance [31]. Therefore, understanding the pathogenesis mechanisms involved in RCC progression is needed to discover potential novel and highly efficient therapies. By establishing a technical platform for micropeptide screening, it was found that the expression of MIAC encoded by lncRNA AC025154.2 was significantly reduced in samples of patients with renal cancer. Endogenous overexpression of MIAC and chemically synthesized MIAC polypeptide treatment could significantly inhibit the growth and metastasis of RCC in experimental models. It was found that anti-tumor effect of MIAC in animal models is superior to current first-line treatment drugs Sunitinib and Axitinib, which may provide a new treatment strategy for kidney cancer. In the future, pharmacodynamics, pharmacokinetic characteristics and early evaluation of MIAC safety can be further explored.

RCC is characterized by a lack of early warning signs and absence of recognized clinical diagnostic markers for its early diagnosis, therefore 20%-30% of RCC patients present at advanced stages, with several cancer metastases at the time of diagnosis [32]. The prognosis of patients with metastatic kidney cancer is poor, and the 5-year survival rate is only 10%-20%. For this reason, finding new diagnostic marker for the detection of RCC before symptom onset enables treatment of less aggressive tumors and provides a better prognosis for patients. Through the analysis and detection of 600 clinical samples of kidney cancer from different sources, this paper proved for the first time that MIAC is low expressed in kidney cancer tissue. Importantly, the expression of MIAC in early patients was significantly higher than that in advanced-stage patients, and it was also significantly related to the prognosis of RCC patients.

Based on our previous results, we conducted co-immunoprecipitation experiment and yeast two hybrid assay to screen the interaction protein of MIAC. In this study, we have identified new interactions involving MIAC and AQP2 protein in RCC. The direct binding of micropeptide MIAC to AQP2 was also confirmed by molecular dynamics simulation, affinity experiment, and Streptavidin pull-down experiment. Accordingly, it was proved that AQP2 is low expressed in renal cancer and can inhibit the proliferation and migration of kidney cancer cells. As mentioned above, AQP2 as a member of the aquaporin family was mainly identified to be involved in the development of congenital nephrogenic diabetes insipidus, diabetes mellitus or heart disease. This study not only provides a reference for the discovery of new targets for kidney cancer treatment, but also broadens the current new understanding of the function of the AQP2 protein. Subsequently, we found that three amino acid residues on the AQP2 protein form the key sites for MIAC binding, but this result need to be verified by other analytical methods like cryo-EM [33] or co-crystallization [34] to accurately determine all the sites of binding between the two molecules in the future. It was also found that AQP2 protein expression is reduced in kidney cancer tissue, however, the number of clinical samples have to be expanded, with combination of liquid biopsy (blood, urine) analysis to explore the application potential of MIAC and AQP2 as biomarkers for kidney cancer diagnosis and prognosis.

**Fig. 7:**
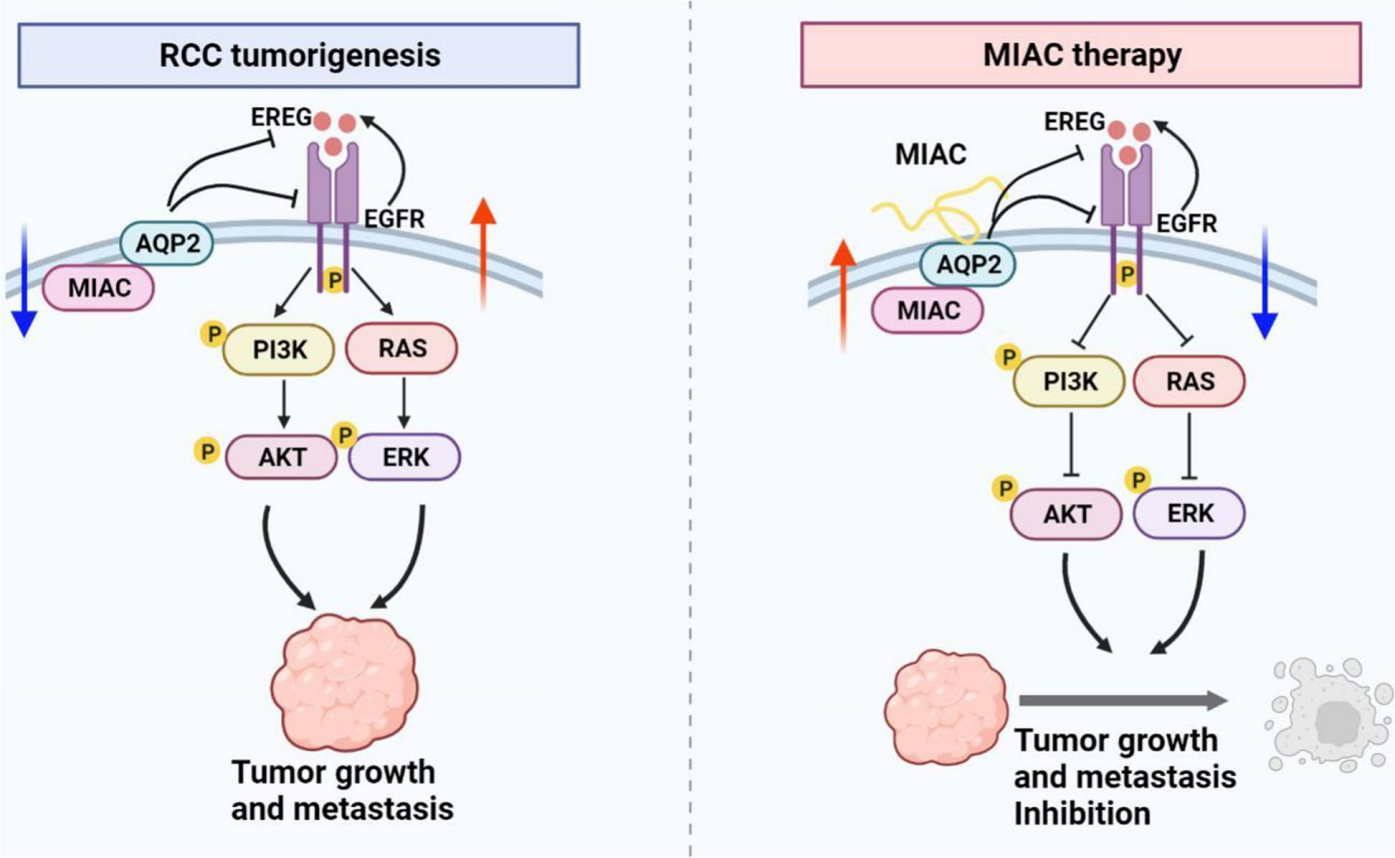
Schematic diagram of the molecular mechanism of MIAC micropeptide inhibiting RCC cell growth and metastasis. MIAC directly binds to AQP2 and suppresses RCC growth and metastasis through inhibiting PI3K/AKT and MAPK downstreams of EREG/EGFR signalling. MIAC has inhibitory effects on RCC in vitro and in vivo, suggesting its potential application in kidney cancer treatment

This study found and confirmed for the first time that MIAC directly binds to AQP2 inhibiting the expression of EREG, EGFR and the activation of downstream PI3K/AKT and mTOR signaling pathways to achieve the inhibitory effect of kidney cancer growth and metastasis (Fig. 7). However, after MIAC direct binding with AQP2, the specific regulatory mechanism to inhibit EREG/EGFR expression and downstream signal pathway activation needs to be further explored. Early high-throughput transcriptome data analysis found that MIAC was differentially expressed in other four tumors, including thyroid cancer, prostate cancer, lung adenocarcinoma, and colon adenocarcinoma. Therefore, in the future, the function and regulatory mechanism of MIAC in other tumors can be explored to evaluate MIAC anticancer spectrum.

## Conclusions

This study revealed for the first time the micropeptide MIAC encoded by lncRNA AC025154.2 is significantly under-expressed in RCC, and had a significant correlation with patient prognosis and tumor stage. And mechanism studies showed that MIAC inhibits the activation of the EREG/EGFR signaling pathway by direct binding to AQP2 protein, thereby inhibiting renal cancer progression and metastasis in vitro and in vivo. In summary, this study not only represents a new advance in elucidating the relationship between micropeptides and tumorigenesis, but also provides new strategies for the diagnosis and treatment of kidney cancer.

## Supplementary Information

**Additional file 1:** **Fig S1. **Knockdown of MIAC promotes RCC cell proliferation and migration. (**A, B**) The relative expression level of MIAC was significantly down-regulated by MIAC knockdown. Knockdown of MIAC promotes RCC cell proliferation (**C**) and cell migration (**D**). (**E**) Knockdown of MIAC promotes RCC cell cycle arrest or inhibits cell apoptosis (**F**). *P<0.05, **P<0.01, ***P<0.001.** Fig S2.** The strategy that explore the specific binding sites between MIAC and AQP2. (**A**) Strategy of recombinant plasmid construction of different AQP2 mutants based on the molecular docking analysis. (**B**) Identify the successful transfection of different AQP2 mutants by GFP fluorescence detection. The purity and molecular weight analysis of chemically synthesized Biotin-MIAC peptide by HPLC (**C**) and LC-MS(**D**).** Fig S3**. Knockdown of AQP2 promotes RCC cell proliferation and migration. (**A**) Relative expression of AQP2 in early and late stage of KIRC samples. (**B**) Kaplan-Meier analysis of the correlation between AQP2 levels and overall survival in RCC samples. (**C, D**) The relative expression level of AQP2 was significantly down-regulated by AQP2 knockdown. Knockdown of AQP2 promotes RCC cell proliferation (**E**) and cell migration (**F**). The RCC cell cycle arrest was promoted by knockdown of AQP2 (**G**). Knockdown of AQP2 has no effect on the RCC cell apoptosis (**H**). *P<0.05, **P<0.01, ***P<0.001, *n.s* no significance** Fig S4**. The purity and molecular weight analysis of chemically synthesized MIAC peptide by HPLC (**A**) and LC-MS (**B**)** Supplementary Table S1.** Clinical pathologic characteristics of 530 KIRC patients in the original TCGA database** Supplementary Table S2. **The sequences of different siRNA** Supplementary Table S3. **The sequences of qPCR primers** Supplementary Table S4. **The molecular docking results of the mainly binding sites between MIAC and AQP2.

## Abbreviations

MIAC: Micropeptide inhibiting actin cytoskeleton
TCGA: The Cancer Genome Atlas
AQP2: Aquaporin 2
sORF: Small open reading frame
ncRNAs: Non-coding RNAs
lncRNA: Long non-coding RNA
circRNA: Circular RNA
miRNA: MicroRNA
UTR: Untranslated region
CRC: Colon cancer
CASIMO1: Cancer-associated small integral membrane open reading frame 1
AML: Acute myeloid leukemia
MPM: Micropeptide in mitochondria
ASAP: ATP synthase-associated peptide
APPLE: A peptide located in ER
HNSCC: Head and neck squamous cell carcinoma
RCC: Renal cell carcinoma
FFPE: Formalin-fixed and paraffin-embedded
qRT-PCR: Quantitative real-time PCR
Co-IP: Co-Immunoprecipitation; CCK-8: Cell Counting Kit-8; BSA: bovine serum albumin; FACS: Flow cytometry analysis
HPLC: High performance liquid chromatography
MST: Microscale thermophoresis
SPR: Surface Plasmon Resonance
