# Supplementary material for: Micropeptide MIAC inhibits the tumor progression by interacting with AQP2 and inhibiting EREG/EGFR signaling in renal cell carcinoma
Source: Mol Cancer. 2022 Sep 19;21:181. doi: 10.1186/s12943-022-01654-1 (PMC9484220; doi:10.1186/s12943-022-01654-1)
Supplement: Supplementary file 1 — Additional file 1: Fig S1. Knockdown of MIAC promotes RCC cell proliferation and migration. (A, B) The relative expression level of MIAC was significantly down-regulated by MIAC knockdown. Knockdown of MIAC promotes RCC cell proliferation (C) and cell migration (D). (E) Knockdown of MIAC promotes RCC cell cycle arrest or inhibits cell apoptosis (F). *P<0.05, **P<0.01, ***P<0.001. Fig S2. The strategy that explore the specific binding sites between MIAC and AQP2. (A) Strategy of recombinant plasmid construction of different AQP2 mutants based on the molecular docking analysis. (B) Identify the successful transfection of different AQP2 mutants by GFP fluorescence detection. The purity and molecular weight analysis of chemically synthesized Biotin-MIAC peptide by HPLC (C) and LC-MS(D). Fig S3. Knockdown of AQP2 promotes RCC cell proliferation and migration. (A) Relative expression of AQP2 in early and late stage of KIRC samples. (B) Kaplan-Meier analysis of the correlation between AQP2 levels and overall survival in RCC samples. (C, D) The relative expression level of AQP2 was significantly down-regulated by AQP2 knockdown. Knockdown of AQP2 promotes RCC cell proliferation (E) and cell migration (F). The RCC cell cycle arrest was promoted by knockdown of AQP2 (G). Knockdown of AQP2 has no effect on the RCC cell apoptosis (H). *P<0.05, **P<0.01, ***P<0.001, n.s no significance Fig S4. The purity and molecular weight analysis of chemically synthesized MIAC peptide by HPLC (A) and LC-MS (B) Supplementary Table S1. Clinical pathologic characteristics of 530 KIRC patients in the original TCGA database Supplementary Table S2. The sequences of different siRNA Supplementary Table S3. The sequences of qPCR primers Supplementary Table S4. The molecular docking results of the mainly binding sites between MIAC and AQP2. [file 12943_2022_1654_MOESM1_ESM.doc]

**Supplementary Materials for**

**Micropeptide MIAC inhibits the tumor progression by**

**interacting with AQP2 and inhibiting EREG/EGFR signaling**

**in renal cell carcinoma**

**This file includes:**

**Fig. S1 to S4**

**Table S1 to S4**

**Fig S1.** Knockdown of MIAC promotes RCC cell proliferation and migration. (**A, B**) The relative expression level of MIAC was significantly down-regulated by MIAC knockdown. Knockdown of MIAC promotes RCC cell proliferation (**C**) and cell migration (**D**). (**E**) Knockdown of MIAC promotes RCC cell cycle arrest or inhibits cell apoptosis (**F**). **P*<0.05, ***P*<0.01, ****P*<0.001.

**Fig S2**. The strategy that explore the specific binding sites between MIAC and AQP2. (**A**) Strategy of recombinant plasmid construction of different AQP2 mutants based on the molecular docking analysis. (**B**) Identify the successful transfection of different AQP2 mutants by GFP fluorescence detection. The purity and molecular weight analysis of chemically synthesized Biotin-MIAC peptide by HPLC (**C**) and LC-MS(**D**).

**Fig S3**. Knockdown of AQP2 promotes RCC cell proliferation and migration. (**A**) Relative expression of AQP2 in early and late stage of KIRC samples. (**B**) Kaplan-Meier analysis of the correlation between AQP2 levels and overall survival in RCC samples. (**C, D**) The relative expression level of AQP2 was significantly down-regulated by AQP2 knockdown. Knockdown of AQP2 promotes RCC cell proliferation (**E**) and cell migration (**F**). The RCC cell cycle arrest was promoted by knockdown of AQP2 (**G**). Knockdown of AQP2 has no effect on the RCC cell apoptosis (**H**). **P*<0.05, ***P*<0.01, ****P*<0.001, *n.s* no significance.

**Fig S4**. The purity and molecular weight analysis of chemically synthesized MIAC peptide by HPLC (**A**) and LC-MS (**B**).

**Supplementary Table S1.** Clinical pathologic characteristics of 530 KIRC patients in

the original TCGA database.

| Characteristic | TCGA cohort (n = 530) |
| --- | --- |
| Age (years) |  |
| Median | 61 |
| Range | 27-90 |
| Gender |  |
| Female | 178 (33.6%) |
| Male | 352 (66.4%) |
| Vital status |  |
| Living | 365 (68.9%) |
| Dead | 165 (31.1%) |
| OS time(days) |  |
| Median | 1177 |
| Range | 0-4537 |
| Clinical stage |  |
| Stage I- II | 324 (61.1%) |
| Stage III- IV | 203 (38.3%) |
| not reported | 3 (0.6%) |

Supplementary Table S2. The sequences of different siRNA.

| Gene name | Sense (5’-3’) | Antisense (5’-3’) |
| --- | --- | --- |
| STK32C | GGCUGUACAUCUGCGAGAUTT | AUCUCGCAGAUGUACAGCCTT |
| SLC9A2 | GCCUGUGUUUACGCUGGAUTT | AUCCAGCGUAAACACGGCTT |
| TAS2R10 | UCCGGUAACCUAAUUGAAUTT | AUUCAAUUAGGUUACCGGATT |
| SPDYC | GCACCAGAGGGAUAAGCUUTT | AAGCUUAUCCCUCUGGUGCTT |
| TTPA | GCUGCUGUACUUACGGAUUTT | AAUCCGUAAGUACAGCAGCTT |
| ONECUT1 | CCAGCGUCGAACUCUACAUTT | AUGUAGAGUUCGACGCUGGTT |
| EREG | CCUCAGUACAACUGUGAUUTT | AAUCACAGUUGUACUGAGGTT |
| TAS2R16 | GCUUGAGUCCUUGACAAUUTT | AAUUGUCAAGGACUCAAGCTT |
| EGFR | GUCCGCAAGUGUAAGAAGUTT | ACUUCUUACACUUGCGGACTT |
| ATP11B | GCUAAGAACUCUGUGUAUATT | UAUACACAGAGUUCUUAGCTT |
| LIMK1 | GACCGGAUCUUGGAAAUCATT | UGAUUUCCAAGAUCCGGUCTT |
| SLFN13 | GCAGACCAGUGUAUUCUAATT | UUAGAAUACACUGGUCUGCTT |
| OAS1 | CCGCAUGCAAAUCAACCAUTT | AUGGUUGAUUUGCAUGCGGTT |
| IFI27 | GACUCUCCGGAUUGACCAATT | UUGGUCAAUCCGGAGAGUCTT |
| PTCD3 | GCAACAGUAUGUGCGAUAATT | UUAUCGCACAUACUGUUGCTT |
| BRINP2 | GCUGCACCAUAUCCAGAUATT | UAUCUGGAUAUGGUGCAGCTT |
| DGKQ | GCAGAUGAGUAACUACUGUTT | ACAGUAGUUACUCAUCUGCTT |
| Negative control | UUCUCCGAACGUGUCACGUTT | ACGUGACACGUUCGGAGAATT |

Supplementary Table S3. The sequences of qPCR primers.

| Primer Name | Forward (5’-3’) | Reverse (5’-3’) |
| --- | --- | --- |
| GAPDH | TGCACCACCAACTGCTTAGC | GGCATGGACTGTGGTCATGAG |
| MIAC | ATGGAGAGAGCAGGGGTGCCCG | CGCCTCTTCTCTGTCCCCAGCTC |
| AQP2 | CTGTGTTCGCAGAGTTCCT | GCCAATACCCAAGCCAAAC |
| TAS2R16 | CCATGGAGCATCTACCAAGAAA | CCAGGAACAGGATGAAAGGAATA |
| SPDYC | CTCAGGCTATATAAGCGCACTC | TCTACCTGGGTGGTAACTACAG |
| LIMK1 | CATGAGCCCAGATGTGAAGAA | CCTGAATCAGCAGGTCAATCTC |
| EREG | GCTCCTTCATCGAATGCTAAAC | CCTAGCACATGCCAGGATAAA |
| EGFR | CCATCCTGGAGAAAGGAGAAC | GCGACTATCTGCGTCTATCATC |
| OAS1 | CGTGTTTCCGCATGCAAAT | TTACCACCTTGGACACACAC |
| IF127 | CTGTCATTGCGAGGTTCTACT | ATTTGGGATAGTTGGCTCCTC |
| DGKQ | TAAGGTGCACATGCTGAGG | CCTACCTAGGATCGCTCTCA |
| SLFN13 | CCAGAGTTTCCTCCAGACTTT | GCTTTGTGTTCCAGACCTTTC |
| SLC9A2 | CGCCAACTTCTTTGTTGTGG | GGCTCGATCACTCGGATATTATG |
| TAS2R10 | AGTTAGTGAGTCAGTGTTTGGG | AGCTAAGCCGGTGAGAATAAAG |
| PTCD3 | TCAAAGATGGCGGTTGTATCT | GAGGGTTGCACTACCAGAATAA |
| BRINP2 | CAGTCAGTTCTGTCTTGGTACA | AAAGCGCTCACGCAGATA |
| STK32C | CTTGAGCTGCTTTGGAGACT | GAGCCTGAGGTGTGAAATGT |
| TTPA | CGGAATGGAATCAAGGCTATCT | CCGTAAGTACAGCAGCAATCT |
| ONECUT1 | CCACAGTCTCGGACAAGTTC | AAGCTACCGCTCACGTTG |

**Supplementary Table S4.** The molecular docking results of the mainly binding sites

between MIAC and AQP2

| Protein | Peptide | Binding Energy（kcal/mol） | Contact Sites（protein） | Contact Sites（Peptide） | Combination Type |
| --- | --- | --- | --- | --- | --- |
| AQP2 | MIAC | -260.81 | GLY-78  CYS-79  TYR-221  LEU-217  GLU-232 | TRP-45  LEU-41  SER-44  ARG-48  ARG-49 | Salt bridge, Hydrogen bond |
